# Unlabeled NMR Approach with Site-Specific Methyl Assignments for Structural Evaluation of the IgG1 Fc Region

**DOI:** 10.1021/jacs.5c18997

**Published:** 2026-02-11

**Authors:** Saeko Yanaka, Yuuki Koseki, Yohei Miyanoiri, Toshio Yamazaki, Tsutomu Terauchi, Daichi Kaneko, Yukiko Isono, Kohei Tomita, Sachiko Kondo, Masayoshi Onitsuka, Maho Yagi-Utsumi, Hirokazu Yagi, Akiko Ishii-Watabe, Koichi Kato

**Affiliations:** † Exploratory Research Center on Life and Living Systems (ExCELLS), National Institutes of Natural Sciences, 5-1 Myodaiji, Okazaki, Aichi 444-8787, Japan; ‡ Institute for Molecular Science (IMS), National Institutes of Natural Sciences, 5-1 Myodaiji, Okazaki, Aichi 444-8787, Japan; § Core for Spin Life Sciences, Okazaki Collaborative Platform, National Institutes of Natural Sciences, 5-1 Higashiyama, Myodaiji, Okazaki 444-8787, Japan; ∥ Materials and Structures Laboratory, Institute of Integrated Research, Institute of Science Tokyo, 4259 Nagastuta-cho, Yokoyama, Kanagawa 226-8503, Japan; ⊥ Graduate School of Materials and Chemical Technology, Department of Materials Science and Engineering, Institute of Science Tokyo, 4259 Nagastuta-cho, Yokoyama, Kanagawa 226-8503, Japan; # Institute for Protein Research, 13013 Osaka University, 3-2 Yamadaoka, Suita, Osaka 565-0871, Japan; g NMR facility, Division of Structural and Synthetic Biology, Center for Life Science Technologies, RIKEN, 1-7-22 Suehiro-cho, Tsurumi-ku, Yokohama City, Kanagawa 230-0045, Japan; h Taiyo Nippon Sanso Corporation, SI Innovation Center, 2008-2 Wada, Tama, Tokyo 206-0001, Japan; i Faculty and Graduate School of Pharmaceutical Sciences, 12963 Nagoya City University, 3-1 Tanabe-dori, Mizuho-ku, Nagoya, Aichi 467-8603, Japan; j Graduate School of Technology, Industrial and Social Sciences, 13109 Tokushima University, Tokushima 770-8513, Japan; k Division of Biological Chemistry and Biologicals, National Institute of Health Sciences, 3-25-26 Tonomachi, Kawasaki-ku, Kawasaki 210-9501, Japan

## Abstract

Monoclonal antibodies are the cornerstone biopharmaceuticals whose safety and efficacy critically depend on their higher-order structure (HOS). Nuclear magnetic resonance (NMR) spectroscopy has emerged as a promising tool for HOS evaluation, yet its application has largely relied on fingerprinting approaches without residue-level interpretation. Here, we report site-specific assignments of methyl resonances in the Fc region of human IgG1, established through amino acid-selective labeling and correlation with backbone resonances using scalar coupling and NOE connectivities, further supported by mutagenesis. These assignments allowed us to identify glycoform-dependent spectral variations, including distinct signatures of core fucosylation and terminal galactosylation, as well as an Fc-specific amino acid substitution. Importantly, these spectral probes were detectable even in antibodies at natural isotopic abundance, enabling practical applications to therapeutic products without isotopic labeling. Furthermore, dynamic filtering highlighted methyl resonances from hinge and receptor-binding residues with elevated mobility, providing localized insights into functional sites. Collectively, our results establish unlabeled methyl NMR as a robust platform for sensitive and practical HOS assessment of therapeutic antibodies. This approach is broadly applicable to monitor glycosylation heterogeneity, chemical modifications, and batch-to-batch consistency, thereby offering a valuable framework for development and quality control of both innovative biopharmaceuticals and biosimilars.

## Graphical abstract

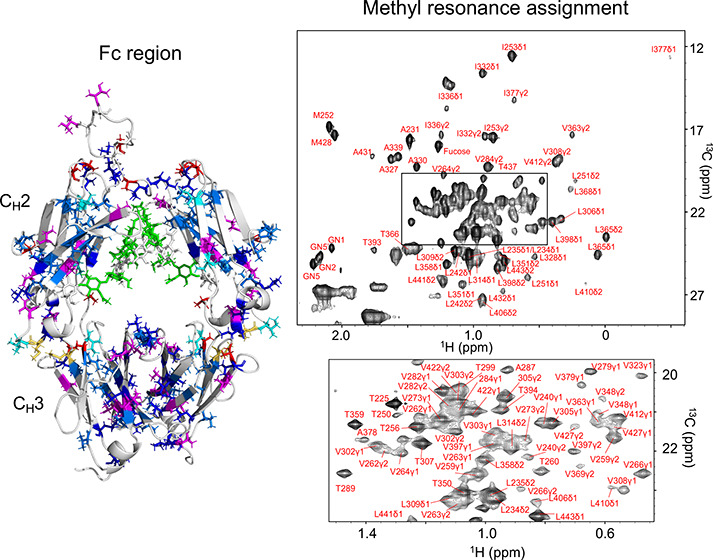

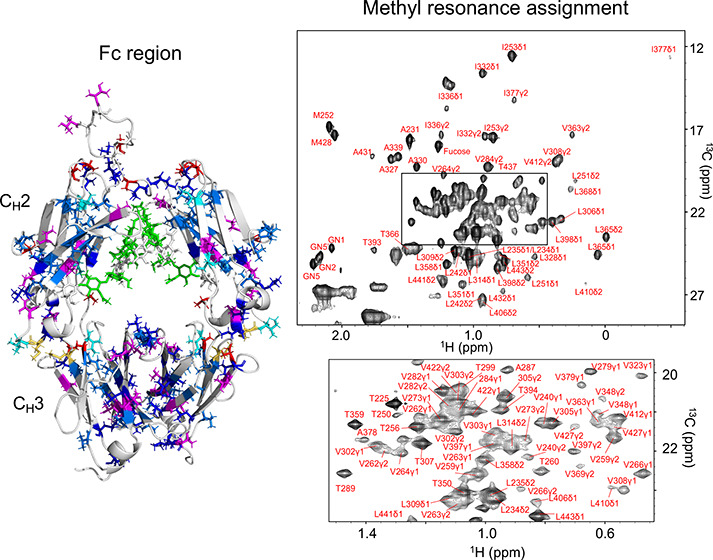

## Introduction

Monoclonal antibody (mAb) therapeutics play a pivotal role in the treatment of major diseases, including cancer, autoimmune disorders, and infectious diseases, and have become central to the global biopharmaceutical market.^1,2^ Recombinant production in mammalian cells, particularly Chinese hamster ovary (CHO) cells, is the mainstream approach for generating high-yield, high-quality antibodies.^3^ At the same time, alternative low-cost expression systems based on yeast, bacteria, or plant cells are actively being explored.^4^ In parallel, antibody engineering has advanced the development of molecules with enhanced functionality, including increased affinity, humanization, and bispecificity, and antibody–drug conjugates (ADCs) that enable targeted delivery of cytotoxic agents.^5,6^

The safety and efficacy of antibody therapeutics are inherently dependent on their higher-order structure (HOS). Therefore, ensuring structural stability under high-concentration formulations and stress conditions during transport, as well as confirming structural consistency across manufacturing batches, is a critical challenge.^7,8^ Biosimilars, developed as alternatives to originator biologics after patent expiration, require rigorous HOS-based comparability study to ensure structural similarity.

Conventional techniques for HOS evaluation include spectroscopic methods such as circular dichroism (CD), Fourier-transform infrared spectroscopy (FT-IR), and fluorescence, thermodynamic analyses using differential scanning calorimetry (DSC), and aggregation/degradation assessment by size-exclusion chromatography.^7,9^ While these ensemble techniques provide valuable global information, they lack residue-level resolution. In recent years, nuclear magnetic resonance (NMR) spectroscopy has emerged as a promising tool for antibody HOS evaluation.^10,11^ NMR potentially provides atomic-level insights into antibody conformation and dynamics in solution, even at formulation-relevant concentrations.

Among NMR techniques, two-dimensional ^1^H–^13^C methyl correlation spectroscopy has proven particularly powerful, enabling HOS evaluation in high-concentration antibody samples and assessment of biosimilar comparability.^12–15^ These advances, together with standardization initiatives, have established NMR fingerprinting as a robust and regulatory-relevant approach for therapeutic antibody characterization. However, such analyses have so far relied mainly on fingerprinting approaches, including principal component analysis (PCA) and clustering, which compress multivariate spectral data into simplified representations of structural similarity.^13^ While effective for manufacturing control and comparability assessments, these approaches do not reveal the molecular basis of structural variations.

To gain mechanistic insights into structural modifications and intermolecular interactions, residue-specific signal assignment is required. Site-specific information is precisely where NMR excels. In this study, we sought to expand the utility of methyl correlation NMR for antibody HOS evaluation by establishing site-specific assignments of ^1^H–^13^C methyl signals in human IgG1, with a particular focus on the Fc region.

The Fc region plays a critical role in immune effector functions through interactions with Fcγ receptors (FcγRs) and complement component C1q.^16^ Fc-fusion proteins, in which therapeutic proteins or peptides are fused with the Fc domain, further highlight the importance of this region by improving half-life, formulation stability, and immune modulation. The Fc region is modified by a conserved *N*-linked glycan whose structure varies depending on the production method and critically influences antibody efficacy.^17,18^ Controlling Fc glycosylation is thus a major focus in therapeutic antibody design and development.

We have previously established stable isotope labeling techniques for the Fc region of IgG1 using various expression systems.^19–21^ We herein employed metabolic labeling in cultured mammalian cells to selectively observe methyl signals from specific amino acids and achieved their unambiguous assignments. As post-translational modifications may vary with production conditions, nonlabeled analysis applicable to formulated antibodies is essential for practical quality assessment. We then evaluated the utility of these site-specifically assigned signals as effective probes for HOS analysis of therapeutic antibodies, even at natural isotopic abundance, thereby establishing a practical platform for sensitive higher-order structure evaluation of therapeutic antibodies in biopharmaceutical development and quality control.

## Results and Discussion

### Fab and Fc-Specific Assignment

We analyzed three therapeutic antibodies widely used in cancer treatment: rituximab (anti-CD20 chimeric IgG1), trastuzumab (anti-HER2 humanized IgG1), and mogamulizumab (anti-CCR4 humanized IgG1), all of which are based on human IgG1 Fc sequences, with trastuzumab differing by allotypic substitution (L358M).^22^ To distinguish Fab- and Fc-derived spectral contributions, we enzymatically fragmented each antibody and compared the methyl spectra of intact IgG molecules with those of the corresponding Fab and Fc fragments. Two-dimensional ^1^H–^13^C methyl correlation spectra were acquired using XL-ALSOFAST-HMQC^23^ pulse sequence (Figure 1; Supplementary Figure 1 and 2). High quality spectra were successfully obtained on an 800 MHz NMR spectrometer equipped with a cryogenic probe within 14 h. The spectra of full-length IgGs were consistent with the additive spectra of their respective fragments, enabling straightforward assignment of Fab- and Fc-derived methyl signals. Whereas Fab spectra varied substantially among the three antibodies, Fc spectra exhibited largely similar patterns, although notable differences were also detected, as described below.

**Figure 1:**
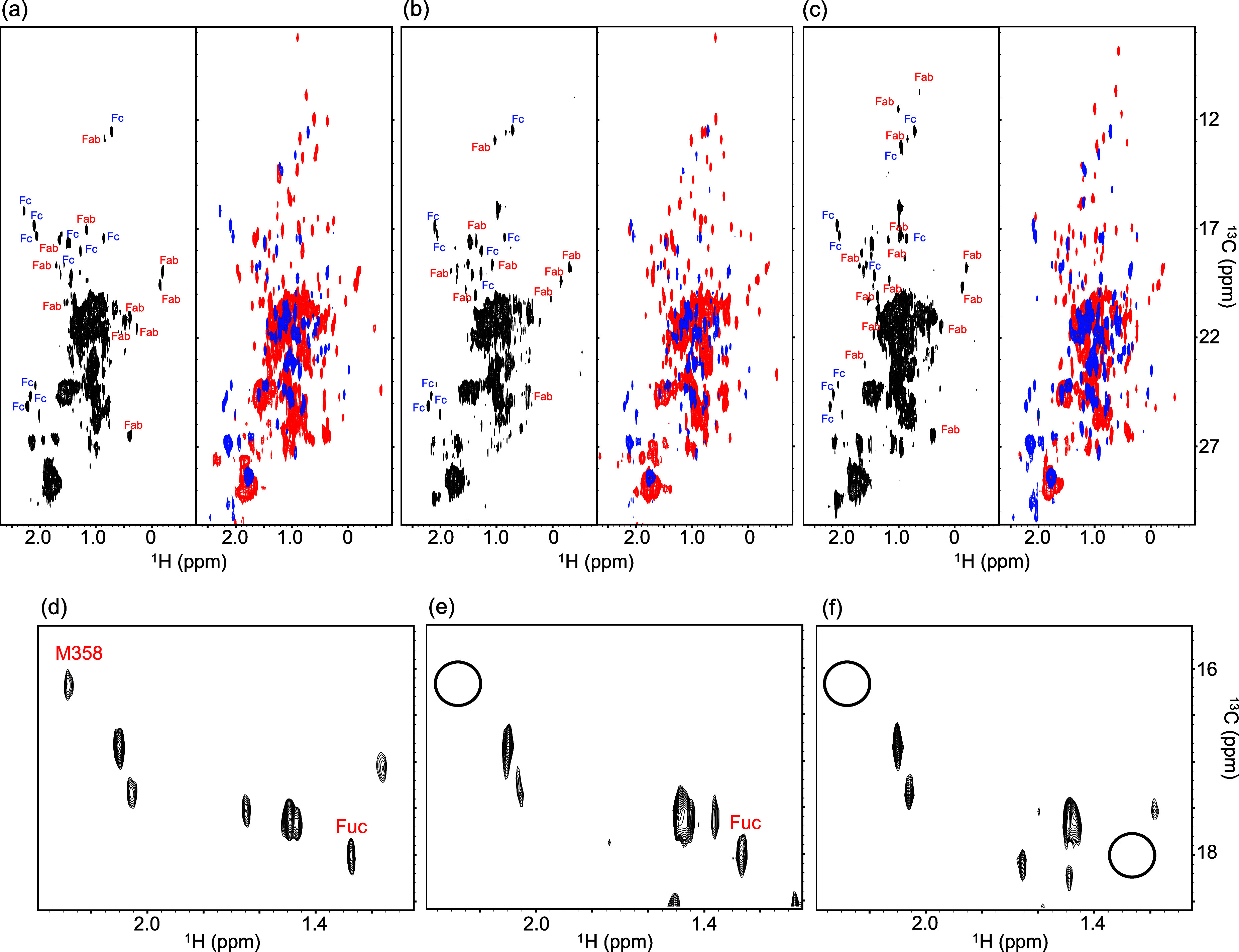
XL-ALSOFAST-HMQC spectra of unlabeled therapeutic antibodies. Panels (a–c) show 2D ^1^H–^13^C methyl correlation spectra of (a) trastuzumab, (b) rituximab, and (c) mogamulizumab. For each antibody, the left subpanel presents the spectrum of the full-length IgG, whereas the right subpanel overlays spectra of the Fab (red) and Fc (blue) fragments, which were used to assign the Fab and Fc signals in the full-length spectrum. Panels (d–f) display enlarged views of the left subpanels in (a–c), respectively. Positions of peaks observed in the reference spectrum but absent in the compared spectrum are indicated by circles. Spectra were recorded at 800 MHz for ^1^H resonance.

### Signal Classification via Amino Acid-Selective Labeling

To classify methyl signals according to amino acid type, we employed metabolic labeling strategies targeting individual residues. Building on our previously established isotope-labeling system for rituximab in CHO cells, which enabled backbone assignments, we here focused on side-chain methyl groups.

As previously demonstrated by Yamaguchi et al., uniformly ^13^C-labeled glucose serves as a metabolic precursor that introduces ^13^C into both glycans and amino-acid residues such as alanine.^24^ In this study, we employed this approach to enable simultaneous observation of Ala methyl signals together with those of fucose and GlcNAc (Figure 2a). The assignments of carbohydrate-derived methyl resonances have been reported previously.^25^ Selective incorporation of ^13^C-labeled threonine provided clear identification of Thr methyl signals (Figure 2b). Likewise, selective ^13^C labeling enabled identification of peaks from Ile residues, with clear discrimination between the two methyl carbons (Cγ2 and Cδ1) due to their distinct chemical shifts (Figure 2c).

**Figure 2:**
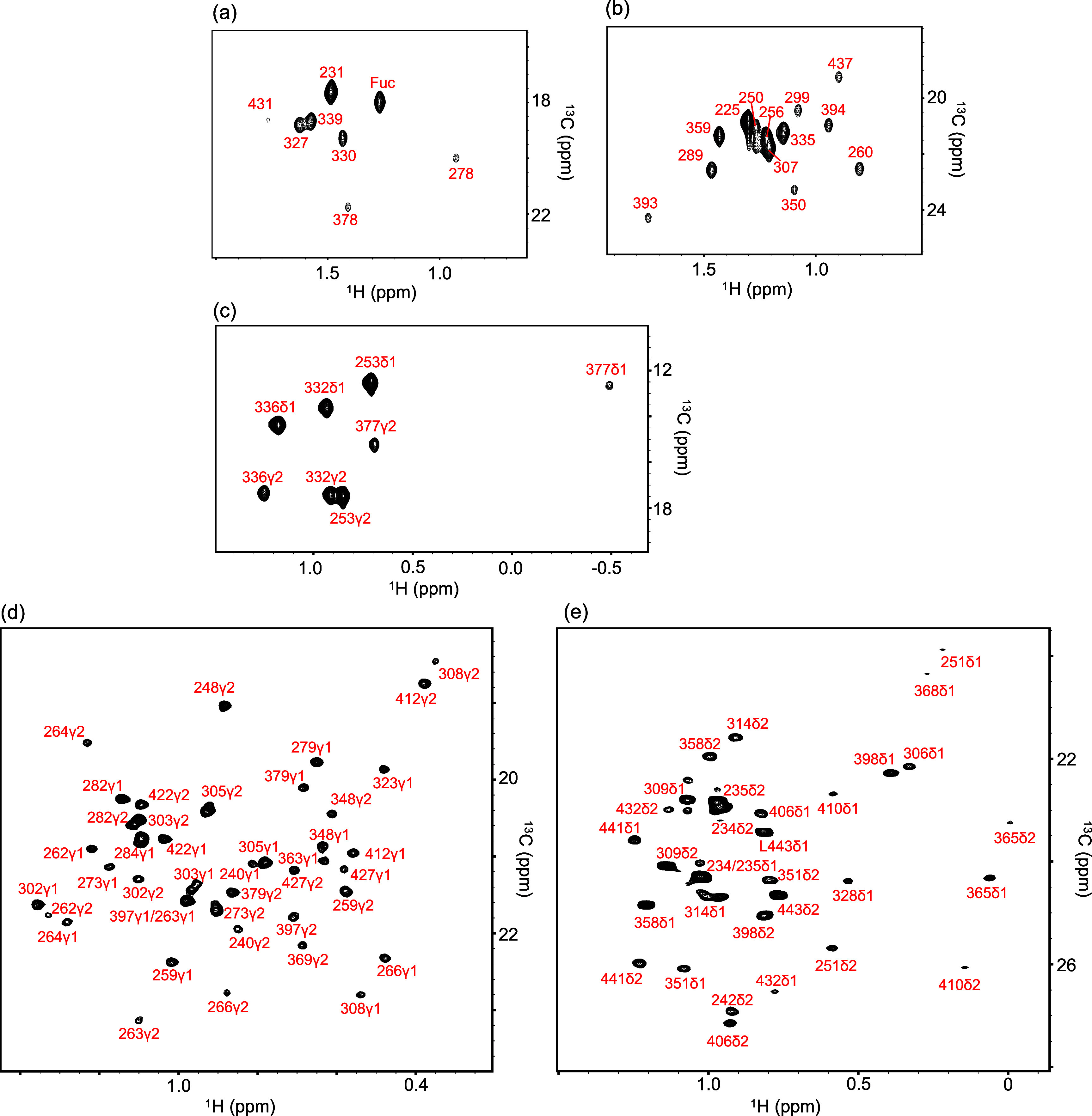
Spectra of amino acid–selectively labeled rituximab Fc. NMR spectra were acquired for rituximab Fc fragments selectively labeled with individual amino acids: (a) Ala, (b) Thr, (c) Ile, (d) Val, and (e) Leu by Methyl-TROSY. The fucose methyl signal appears in the region overlapping with the Ala methyl resonances. Panels (a–c) were recorded at 800 MHz for ^1^H resonance using XL-ALSOFAST-HMQC; panels (d–e) were recorded at 950 MHz for ^1^H resonance using methyl-TROSY.

Leucine and valine, each possessing two methyl groups with similar chemical environments, required tailored strategies. To selectively observe Val methyl signals at high resolution using methyl-TROSY,^26^ we used valine stereospecifically ^13^C-labeled at the Cγ1 position for metabolic incorporation (Supplementary Figure 3a). For simultaneous detection of both Val methyl groups, we applied a dual-isotopomer approach using an equimolar mixture of two selectively labeled valine isotopomers: one labeled at Cγ1 and the other at Cγ2, with all remaining positions deuterated. This strategy suppressed dipolar relaxation and yielded sharp, well-resolved signals for both Val methyl groups (Figure 2d). An analogous approach was successfully applied to Leu residues, allowing selective detection and differentiation of Cδ1 and Cδ2 signals (Figure 2e).

Rituximab Fc also contains two Met residues, for which selective labeling and assignments had been previously established.^43^ Taken together, these labeling strategies enabled comprehensive classification of rituximab Fc methyl signals by residue type.

### Site-Specific Assignments

To achieve site-specific assignments of Fc methyl signals, we employed two complementary approaches. First, assignments were extended from backbone resonances using scalar and NOE connectivities, exemplified by the identification of Ala residues (Supplementary Figure 3c). Second, site-directed mutagenesis combined with amino acid–selective labeling enabled unambiguous confirmation of methyl peaks, as demonstrated by the assignment of Leu398 through the L398V substitution (Supplementary Figure 3d).

By integrating these strategies, we achieved complete assignment of methyl resonances in rituximab Fc. The resulting assignments were mapped onto the spectrum of the unlabeled Fc fragment (Figure 3) and visualized on the crystal structure (Figure 4). These site-specific methyl probes provide a sensitive means to detect subtle structural changes within the Fc region.

**Figure 3:**
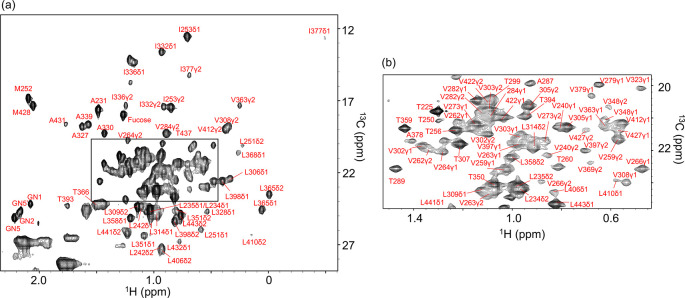
Comprehensive methyl signal assignments for Ala, Ile, Leu, Met, Thr, Val, and GlcNAc residues in unlabeled rituximab Fc. (a) Spectrum covering the entire methyl region and (b) enlarged view of the crowded region of unlabeled rituximab Fc. Spectra were acquired at 800 MHz for ^1^H resonance using XL-ALSOFAST-HMQC.

**Figure 4:**
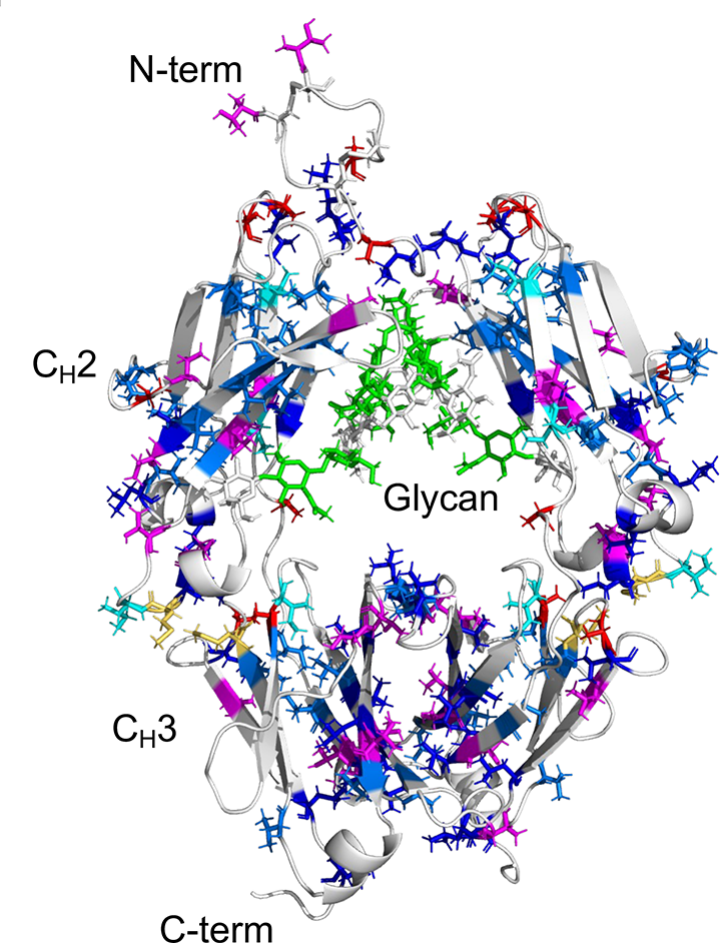
Locations of methyl-bearing residues in the IgG1-Fc structure. Amino acid residues containing methyl groups are colored as follows: Ala (red), Thr (magenta), Met (yellow), Leu (blue), Val (marine blue), Ile (cyan), and GlcNAc (green), in a 3D model constructed in our previous study^44^ based on the crystal structure of IgG1-Fc (PDB ID: 3AVE).

### Utility of Methyl Signals As Structural Probes

To assess the structural impact of Fc glycan heterogeneity, we compared methyl spectra among glycoengineered Fc fragments and commercial antibodies. The conserved *N*-glycosylation site at Asn297 typically carries complex biantennary glycans that vary at their nonreducing termini, particularly in galactosylation and fucosylation. Rituximab Fc fragments with defined glycoforms were prepared using a previously established system based on controlled glycosyltransferase expression and in vitro enzymatic remodeling.^25^ Glycan profiles of four representative variants—FG0, FG2, G0, and G2—were confirmed by HPLC analysis (Supplementary Figure 4).

Spectra acquired at natural isotopic abundance revealed distinct glycoform-dependent differences in the methyl region (Supplementary Figure 5). The fucose methyl resonance present in FG0 was absent in G0, and the GlcNAc2 and V264γ2 methyl groups showed glycoform-specific chemical shift changes (Figure 5a). Whereas glycoform-dependent perturbations of fucose and GlcNAc methyl signals have previously been described only in ^13^C-labeled Fc,^25^ we now show that these features—and the newly identified shift at V264γ2—are detectable even in unlabeled samples, allowing their site-specific interpretation.

**Figure 5:**
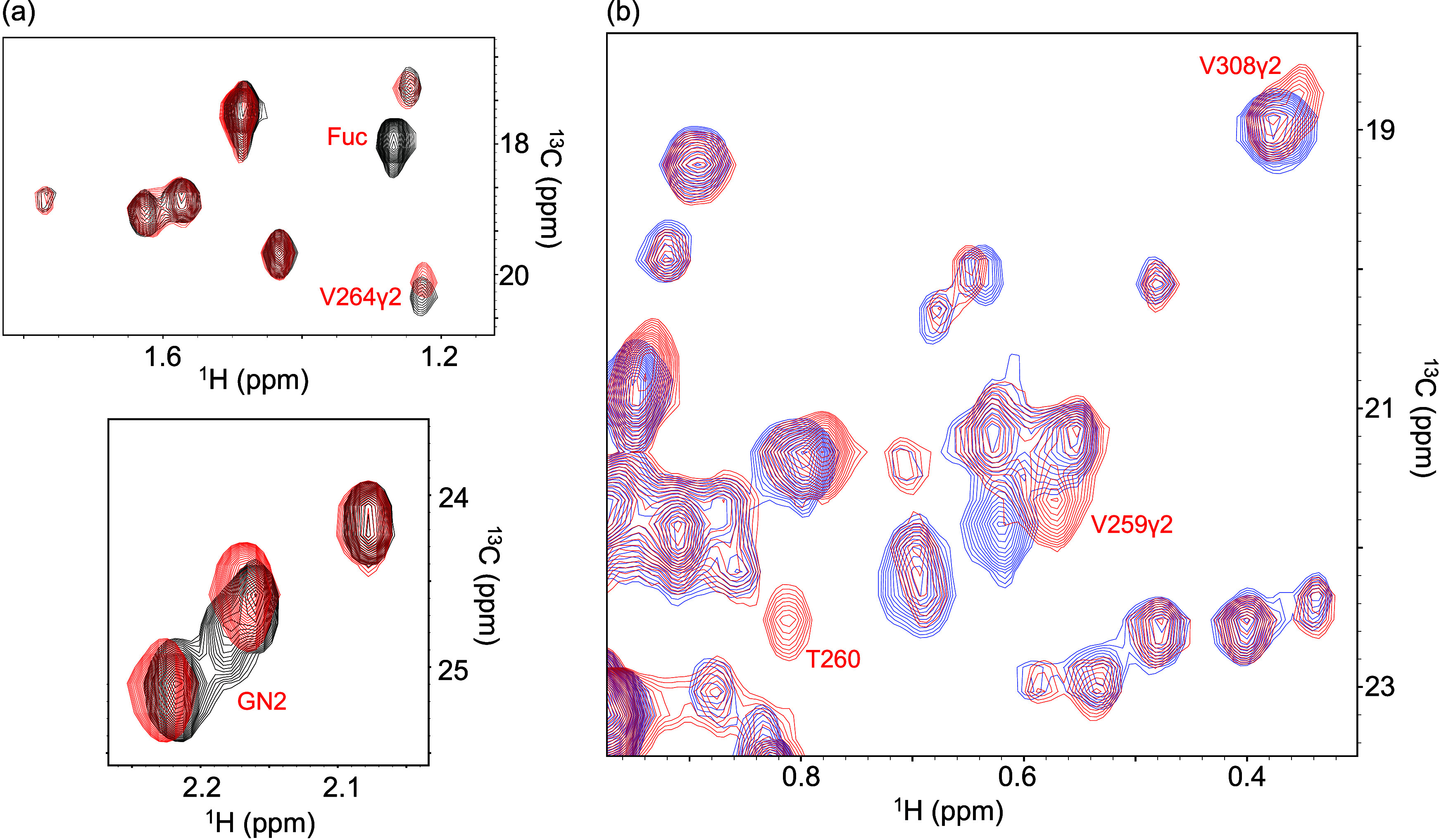
Glycoform-dependent spectral changes in unlabeled rituximab Fc. Expanded regions of XL-ALSOFAST-HMQC spectra comparing (a) FG0 (black) and G0 (red) and (b) G0 (red) and G2 (blue) glycoforms. Spectra were recorded at 800 MHz for ^1^H resonance.

Comparisons between FG0 and FG2 further showed chemical shift changes in Val259 γ2, Thr260, and GlcNAc methyl resonances (Figure 5b). Because the *N*-glycan is embedded within the Fc quaternary structure and the galactose on the Manα1–6 branch interacts with residues such as Thr260, these changes likely reflect microenvironmental perturbations induced by degalactosylation.

Analysis of commercial antibodies demonstrated that rituximab and trastuzumab were fully fucosylated, whereas mogamulizumab lacked core fucose (Supplementary Figure 6), consistent with their NMR spectral features. Remarkably, the fucose methyl signal was detectable even in unlabeled full-length IgG. In addition, the spectrum of trastuzumab exhibited an extra Met resonance compared with the other two antibodies, consistent with its L358M substitution.^22^

### Dynamic Filtering of Methyl Resonances

The intensities of methyl resonances varied markedly even among residues of the same type, reflecting differences in relaxation properties associated with residue-specific internal dynamics. In the Fc region, solvent-exposed residues generally exhibited stronger signals, consistent with higher mobility. In spectra of intact IgG, adjusting the contour threshold highlighted only the most intense resonances, which could be attributed to Thr223, Thr225, Leu234, and Leu235 (Figure 6). This reflects a dynamic filtering effect, which enables the selective observation of signals originating from regions with exceptionally high mobility within large proteins.^27–31^ These Thr residues are located in hinge segments that impart flexibility to the Fab arms, whereas the Leu residues constitute part of the Fcγ receptor–binding site.^32,33^ These residues therefore represent dynamic probes linked to critical IgG functions.

**Figure 6:**
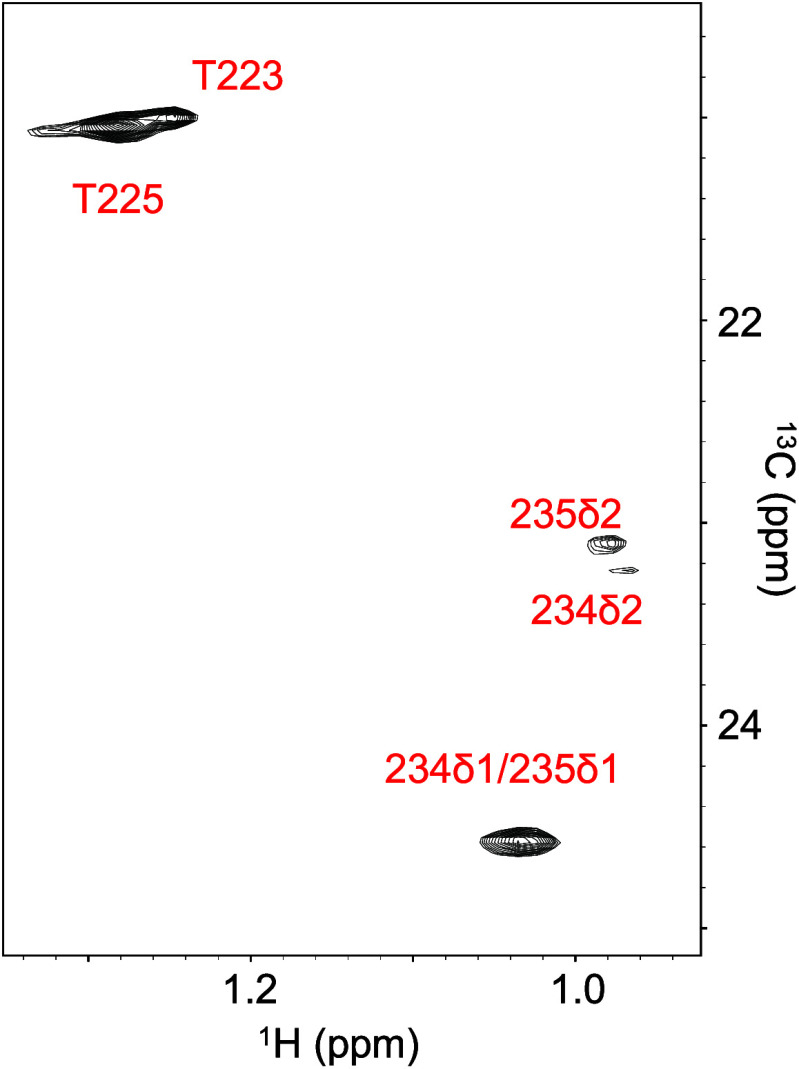
Selective observation of methyl HSQC peaks originating from highly mobile sites of unlabeled rituximab Fc. Strong signals corresponding to γ-methyl groups of Thr223 and Thr225 and δ-methyl groups of Leu234 and Leu235 are observed, allowing selective observation of the hinge residues. Spectra were recorded at 800 MHz for ^1^H resonance.

Although spectra of full-length IgG suffer from substantial signal overlap compared with Fab or Fc fragments, exploiting intensity differences through dynamic filtering provides localized information on functionally important sites. This strategy offers a complementary avenue for “dynamic” higher-order structural evaluation of therapeutic antibodies, providing complementary information on global dynamics from small-angle X-ray scattering, which is recently highlighted for its use in quality control of IgG therapeutics.^34^

## Conclusion and Future Perspectives

This study establishes a robust framework for HOS evaluation of therapeutic antibodies by combining site-specific methyl signal assignments with NMR measurements at natural isotopic abundance. By integrating amino acid–selective labeling, fragment dissection, and correlation with backbone resonances, we established residue-level probes that enable highly sensitive detection of structural variations arising from amino acid substitutions and glycosylation differences, even in unlabeled samples. This strategy revealed subtle yet functionally significant Fc structural differences, including those associated with core fucosylation and terminal galactosylation—two key glycan features known to critically modulate effector functions such as antibody-dependent cellular cytotoxicity and complement activation. In addition to glycan heterogeneity, the approach provides a powerful means to detect microstructural perturbations arising from chemical modifications, including oxidation and deamidation, which represent major pathways of therapeutic antibody instability.

Looking ahead, this methodology offers a versatile platform for evaluating formulation-induced stresses, assessing batch-to-batch consistency, and supporting regulatory review of both innovative biopharmaceuticals and biosimilars. Its inherent adaptability to diverse antibody formats and manufacturing systems underscores its potential as a practical and scalable solution for next-generation biopharmaceutical development and quality assurance.

## Materials and Methods

### Materials

Rituximab-producing CHO cell lines with and without FUT8 knockout were prepared in-house as described previously.^35^ The mammalian cell expression system Expi293 was used to express rituximab Fc mutants. Plasmids of rituximab Fc WT and mutants in pcDNA3.4 vector were transfected into Expi293 cells for expression. The IgGs of biotherapeutic antibodies trastuzumab, mogamulizumab, and rituximab, were purchased from Kyowa Kirin Co. Ltd., Nippon Kayaku Co. Ltd., and Zenyaku Kogyo Co. Ltd., respectively.

### Cell Culture and IgG Preparation

The rituximab producing CHO cells were cultured in BalanCD CHO Growth A medium (Fujifilm wako) to express unlabeled rituximab. For stable isotope labeling, the medium was replaced with Nissui NYSF 404 medium supplemented with isotope-labeled metabolic precursors during the final stage of cell culture. Metabolic ^13^C labeling of IgG1-Fc N-glycans was achieved using a modified Nissui NYSF 404 medium, in which glucose was substituted with 2 g/L of D-[^13^C_6_]­glucose. For amino acid-selective labeling, the following isotope-labeled amino acids were used in place of natural amino acids: L-[U–^13^C,^15^N]­threonine, L-[U–^13^C,^15^N]­isoleucine, L-[δ-^13^CH_3_;2,3,3,4,5,5,5-^2^H7]­leucine (racemic δ-methyl), [δ2-^13^C]­leucine, L-[γ-^13^CH_3_;2,3,4,4,4-^2^H_5_]­Valine (racemic δ-methyl), and L-[γ1-^13^C]­valine.^21^

For expression of rituximab Fc mutants, plasmids of WT and mutants in pcDNA3.4 vector were transfected into Expi293 cells. The Gibco Expi293 Expression System Kit (Thermo Fisher Scientific) and Gxpress 293 Transfection Kit (GMEP, Fukuoka, Japan) were used for protein expression. Transfection and culture condition were performed according to the kit protocol. Labeling of the IgG1-Fc glycoproteins was achieved by cultivating the transfected cells in medium containing the labeled amino acid mixture as previously described. Four days after transfection, collected cells were separated by centrifugation. After cell culturing, the supernatant was subjected to purification using a protein A affinity column (Cytiva), following established protocols.^36^

### Enzymatic Treatments

The Fc fragments of IgGs were prepared by papain digestion performed at 37 °C for 12 h in 75 mM sodium phosphate buffer (pH 7.0) containing 75 mM NaCl, and 2 mM EDTA. The ratio of papain/IgG was 1:50 (w:w). Digestion products were separated into Fab and Fc by a protein A affinity column and/or a Hitrap Q HP anion-exchange column (Cytiva), followed by size-exclusion chromatography on a Superdex 200 Hiload 10/300 GL column (Cytiva). To prepare IgG exhibiting a homogeneous *N*-glycan, the Fc fragment was treated with a recombinant *Streptococcus pneumoniae* β1,4-galactosidase (New England Biolabs) and/or bovine galactosyltransferase (Sigma-Aldrich) according to the literature.^35,37^

### Glycosylation Profiling

*N*-glycosylation profiling was performed using the high-performance liquid chromatography (HPLC) mapping technique as previously described.^38^ IgG (0.1 mg) was used as the starting material. *N*-glycans from IgG glycoproteins were released using PNGase A from almond, labeled with 2-aminopyridine, and loaded onto an octadecylsilyl (ODS) column (Shimadzu Co., Kyoto, Japan). The elution time for each HPLC column was expressed in glucose units (G.U.), referenced to a pyridylamino (PA)-derivatized isomalto-oligosaccharide mixture. Structural identification was based on elution positions in the GALAXY database^39^ (http://www.glycoanalysis.info) and comparisons with known IgG *N*-glycosylation patterns.^40^

### NMR Measurements

IgG and Fc samples (20 mg/mL) were prepared in 5 mM sodium phosphate buffer (pH 7.4) containing 50 mM NaCl and 100% (v/v) D_2_O for ^1^H–^13^C HSQC, XL-ALSOFAST-HMQC, ^1^H–^13^C HMQC, ^13^C-edited NOESY, and HCCH–COSY spectral measurements. or at pH 6.0 with 5% (v/v) D_2_O for ^15^N-edited TOCSY and ^15^N-edited HSQC-NOESY spectral measurements. NMR spectra were acquired at 42 °C on AVANCE III 950 and AVANCE NEO 800 (Bruker BioSpin) spectrometers equipped with cryogenic probes. Two-dimensional ^1^H–^13^C correlation spectra were recorded using XL-ALSOFAST-HMQC, methyl-TROSY, or HSQC pulse sequences. Other details of the measurement conditions are summarized in Supplementary Table 1. Chemical shifts of ^1^H were referenced to DSS (0 ppm), and ^13^C chemical shifts were referenced indirectly using the gyromagnetic ratios of ^13^C, and ^1^H (γ^13^C/γ^1^H = 0.25144952). Assignments of methyl groups were made on the basis of previous backbone assignments,^25,35,41^ and^1^H–^13^C XL-ALSOFAST-HMQC spectral data of ^13^C- and ^15^N-labeled IgG1 (or Fc), together with spectral information obtained from the following experiments: ^13^C-edited NOESY, HCCH–COSY, ^15^N-edited TOCSY, and ^15^N-edited HSQC-NOESY. All NMR data were processed using TopSpin software (Bruker BioSpin), and analyzed with POKY.^42^ The chemical shift assignments for the methyl groups of FG0 were deposited under the accession number 53403.

## Supplementary Material



## Notes

XL-ALSOFAST-HMQC spectra of Fab and Fc fragments of unlabeled therapeutic IgG antibodies (trastuzumab, rituximab, and mogamulizumab); methyl signal assignment procedures based on methyl-TROSY, ^13^C-edited NOESY, and site-directed mutagenesis; HPLC elution profiles of PA-derivatized *N*-linked oligosaccharides from IgG Fc regions with different glycoforms and enzymatic treatments; XL-ALSOFAST-HMQC spectra of rituximab Fc fragments with defined glycoforms; and a supplementary table summarizing experimental data (PDF)
