# Supplementary material for: Unlabeled NMR Approach with Site-Specific Methyl Assignments for Structural Evaluation of the IgG1 Fc Region
Source: J Am Chem Soc. 2026 Feb 11;148(8):8374–82. doi: 10.1021/jacs.5c18997 (PMC12964402; doi:10.1021/jacs.5c18997)
Supplement: Supplementary file 1 [file ja5c18997_si_001.pdf]

Unlabeled NMR Approach with Site-Specific Methyl Assignments for Structural Evaluation of the IgG1 Fc Region

Saeko Yanaka[a,b,c,d,e*], Yuuki Koseki [d,e], Yohei Miyanoiri[a,c,f], Toshio Yamazaki[g], Tsutomu Terauchi[h], Daichi Kaneko[h], Yukiko Isono[a,b], Kohei Tomita[i], Sachiko Kondo[a,i], Masayoshi Onitsuka[j], Maho Yagi-Utsumi[a,b,c,i], Hirokazu Yagi[a,i], Akiko Ishii-Watabe[k], Koichi Kato[a,b,c,i,*]

[a]Exploratory Research Center on Life and Living Systems (ExCELLS), National Institutes of Natural Sciences, 5-1 Myodaiji, Okazaki, Aichi 444-8787, Japan, [b]Institute for Molecular Science (IMS), National Institutes of Natural Sciences, 5-1 Myodaiji, Okazaki, Aichi 444-8787, Japan, [c]Core for Spin Life Sciences, Okazaki Collaborative Platform, National Institutes of Natural Sciences, 5-1 Higashiyama, Myodaiji, Okazaki 444-8787, Japan, [d]Materials and Structures Laboratory, Institute of Integrated Research, Institute of Science Tokyo, 4259 Nagastuta-cho, Yokoyama, Kanagawa 226-8503, Japan, [e]Graduate School of Materials and Chemical Technology, Department of Materials Science and Engineering, Institute of Science Tokyo, 4259 Nagastuta-cho, Yokoyama, Kanagawa 226-8503, Japan, [f]Institute for Protein Research, Osaka University, 3-2 Yamadaoka, Suita, Osaka 565-0871, Japan, [g]NMR facility, Division of Structural and Synthetic Biology, Center for Life Science Technologies, RIKEN, 1-7-22 Suehiro-cho, Tsurumi-ku, Yokohama City, Kanagawa, 230-0045, Japan, [h]Taiyo Nippon Sanso Corporation, SI Innovation Center, 2008-2 Wada, Tama, Tokyo 206-0001, Japan, [i]Faculty and Graduate School of Pharmaceutical Sciences, Nagoya City University, 3-1 Tanabe-dori, Mizuho-ku, Nagoya, Aichi 467-8603, Japan, [j]Graduate School of Technology, Industrial and Social Sciences, Tokushima University, Tokushima 770-8513, Japan, [k]Division of Biological Chemistry and Biologicals, National Institute of Health Sciences, 3-25-26 Tonomachi, Kawasaki-ku, Kawasaki 210-9501, Japan

***Corresponding authors:** Koichi Kato, ph.D. (kkatonmr@ims.ac.jp) and Saeko Yanaka, ph.D (yanaka.s.ab@m.titech.ac.jp)

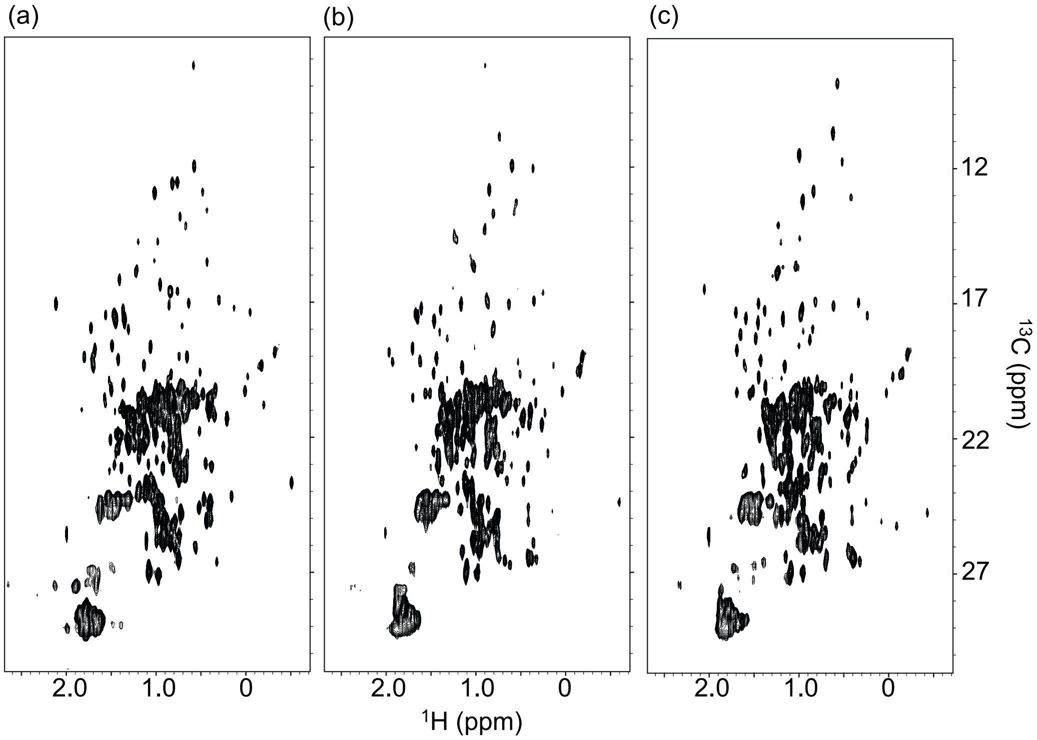

Supplementary Figure 1.

XL-ALSOFAST-HMQC spectra of Fab fragments of unlabeled therapeutic antibodies. Spectra are shown for (a) trastuzumab, (b) rituximab, and (c) mogamulizumab. Spectra were recorded at 800 MHz for ^{1}H resonance.

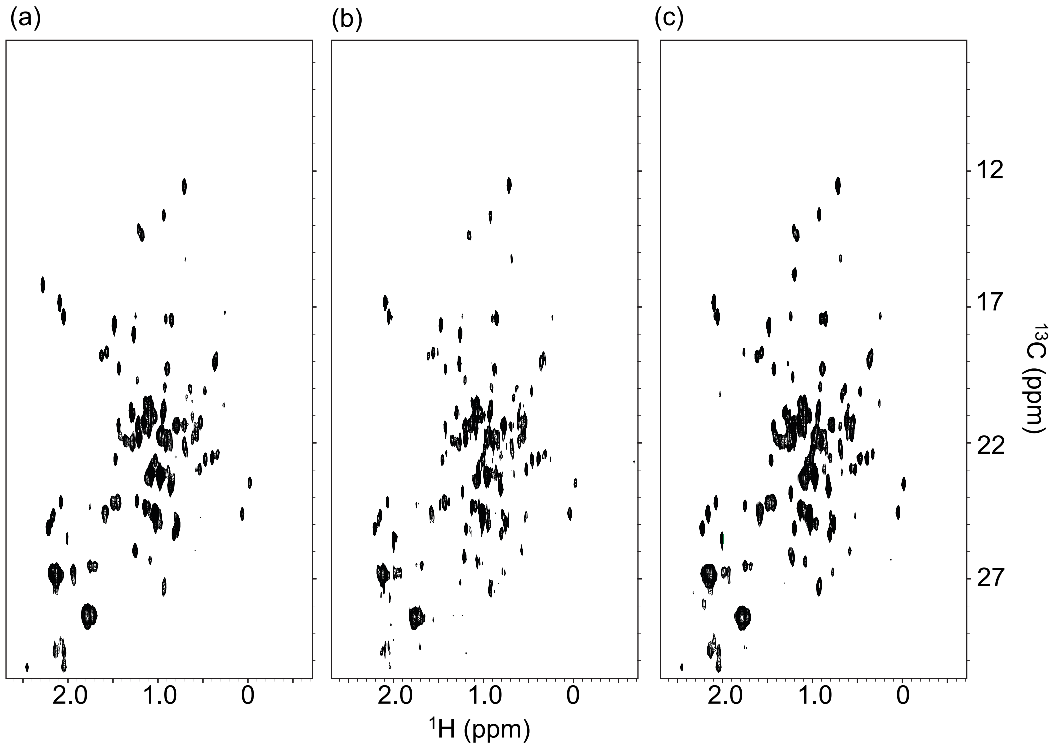

Supplementary Figure 2.

XL-ALSOFAST-HMQC spectra of Fc fragments of unlabeled therapeutic antibodies: (a) trastuzumab, (b) rituximab, and (c) mogamulizumab. Spectra were recorded at 800 MHz for ^{1}H resonance.

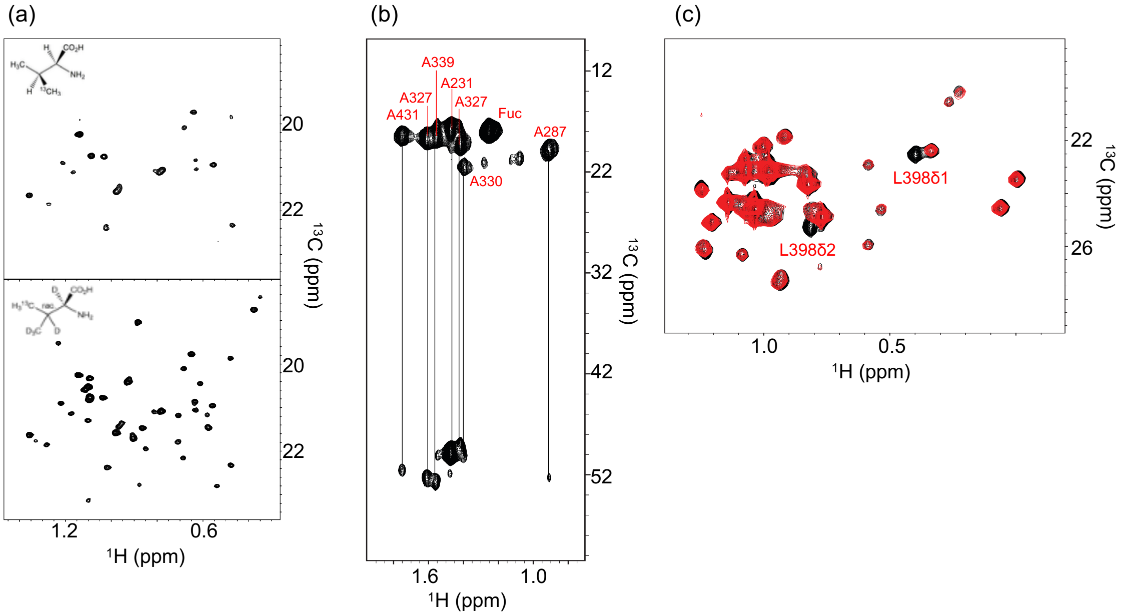

Supplementary Figure 3.

Examples of methyl signal assignment procedures.

(a) Comparison of methyl-TROSY spectra of rituximab Fc selectively labeled with L-[γ1-^{13}C]valine (upper) and L-[γ-$^{13}CH_3$;2,3,4,4,4-$^{2}H_5$]valine (racemic δ-methyl) (lower). Spectra were recorded at 950 MHz for ^{1}H resonance.

(b) ^{13}C-edited NOESY spectrum showing assignment of Ala methyl groups through NOE connectivities with previously assigned Cα protons[1,2]. Spectrum was recorded at 950 MHz for ^{1}H resonance.

(c) Site-directed mutagenesis–based assignment: superposition of ^{1}H–^{13}C HMQC spectra of uniformly ^{13}C-labeled rituximab Fc of the wild type (black) and the L398V mutant (red). Spectra were recorded at 800 MHz for ^{1}H resonance.

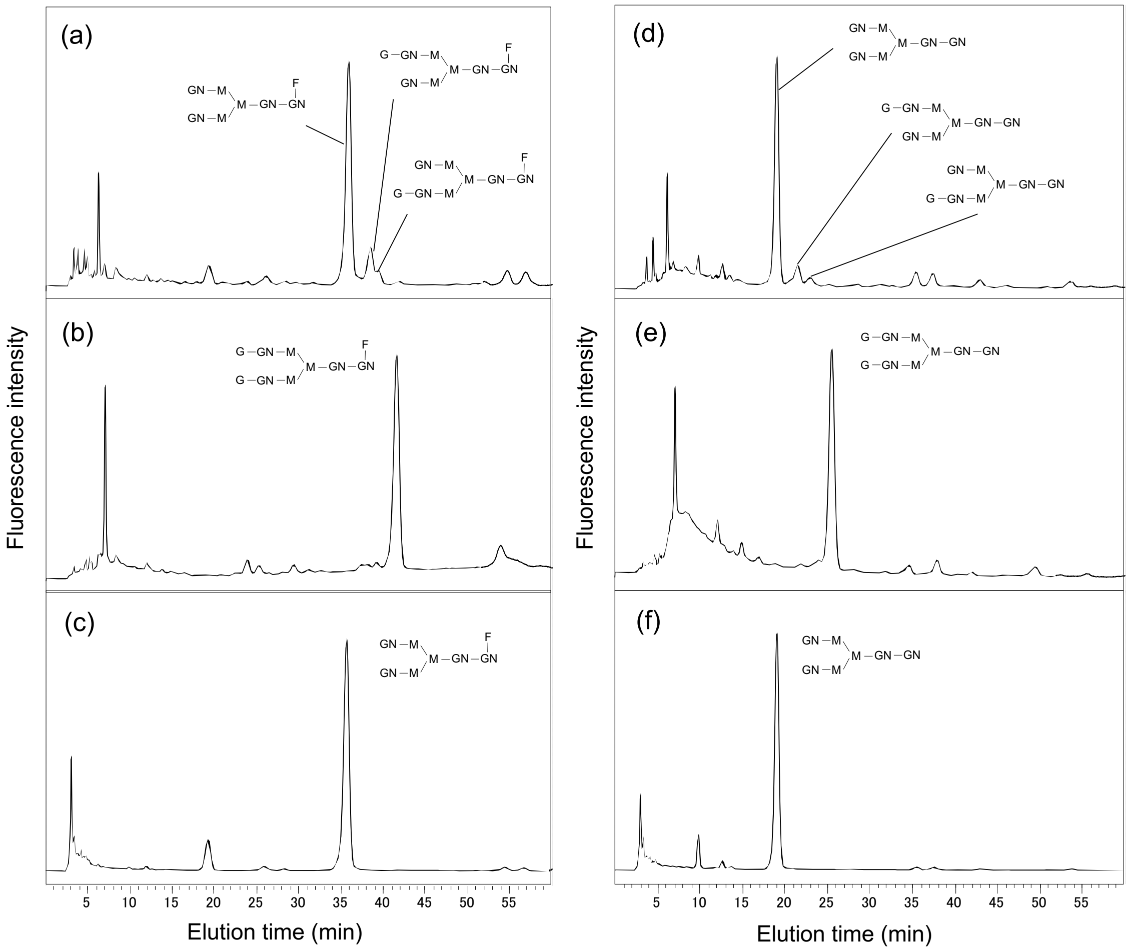

Supplementary Figure 4.

HPLC elution profiles on an ODS column for PA-derivatives of *N*-linked oligosaccharides released from rituximab expressed in CHO cells. Profiles are shown for rituximab (a) without enzymatic treatment, (b) after galactosyltransferase treatment, and (c) after galactosidase treatment, as well as for rituximab expressed in FUT8-knockout cells (d) without enzymatic treatment, (e) after galactosyltransferase treatment, and (f) after galactosidase treatment. One-letter codes are used to indicate the sugar residues as follows: F, fucose; G, galactose; GN, *N*-acetylglucosamine; M, mannose.

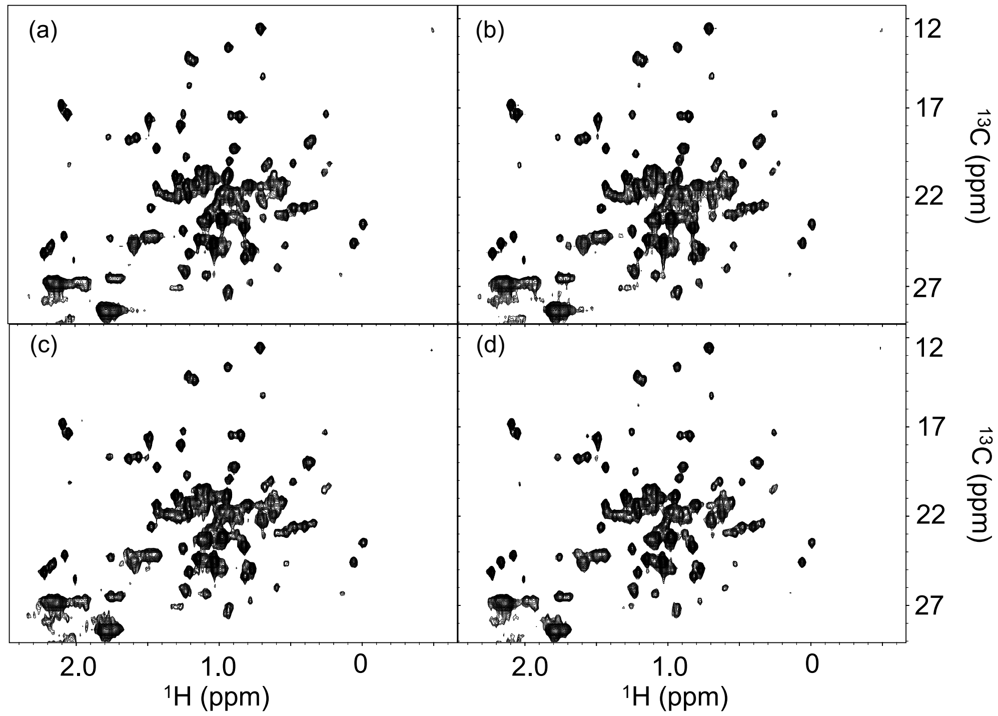

Supplementary Figure 5.

XL-ALSOFAST-HMQC spectra of rituximab Fc fragments with different glycoforms. Spectra are shown for (a) FG0, (b) G0, (c) FG2, and (d) G2 glycoforms. Spectra were recorded at 800 MHz for ^{1}H resonance.

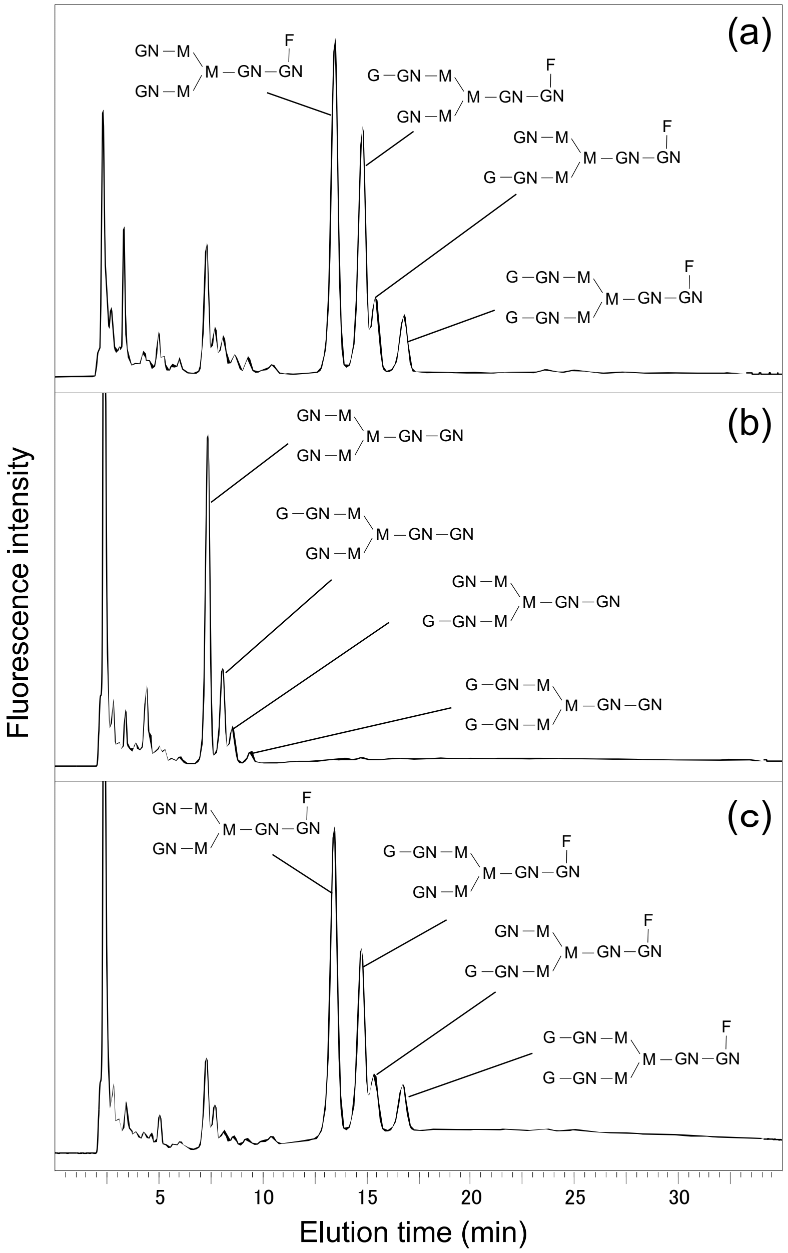

Supplementary Figure 6.

Elution profiles on an ODS column for PA-derivatives of the *N*-linked oligosaccharides released from therapeutic IgGs. Profiles are shown for (a) trastuzumab, (b) mogamulizumab, and (c) rituximab.

Supplementary Table 1. NMR experimental conditions for XL-ALSOFAST-HMQC and methyl-TROSY measurements

Figure(s)	Pulse sequence	Field (MHz)	TD (t_2/t_1)	SW (ppm, $^1H/^{13}C$)	Carrier (ppm, $^1H/^{13}C$)	NS	Relaxation delay (s)
Fig. 1, 2a–c, 3a, 5; Suppl. Fig. 1, 2, 3c, 5	XL-ALSOFAST-HMQC	800	1920/302	12/44	4.7/60	150	1
Fig. 2d; Suppl. Fig. 3a	methyl-TROSY	950	2048/256	16/4	4.7/19.5	16	2
Fig. 2e	methyl-TROSY	950	2048/256	16/12	4.7/22	16	2
Fig. 3b	XL-ALSOFAST-HMQC	800	2048/600	12/20	4.7/20	400	1
Fig. 6	HSQC	800	2048/512	12/25	4.7/17.5	300	1
Suppl. Fig. 3b	HCCH-COSY	800	2048/8/128	14/80/80	4.7/43/43	64	1

Abbreviations: TD, number of complex points in t_2/t_1; SW, spectral width ($^1H/^{13}C$, ppm); Carrier, transmitter frequency for $^1H/^{13}C$ (ppm); NS, number of scans per increment.

References

(1) Yagi, H.; Zhang, Y.; Yagi-Utsumi, M.; Yamaguchi, T.; Iida, S.; Yamaguchi, Y.; Kato, K. Backbone ^{1}H, ^{13}C, and ^{15}N Resonance Assignments of the Fc Fragment of Human Immunoglobulin G Glycoprotein. *Biomol. NMR Assign.* **2015**, *9* (2), 257–260.

(2) Yanaka, S.; Yogo, R.; Yagi, H.; Onitsuka, M.; Wakaizumi, N.; Yamaguchi, Y.; Uchiyama, S.; Kato, K. Negative Interference with Antibody-Dependent Cellular Cytotoxicity Mediated by Rituximab from Its Interactions with Human Serum Proteins. *Front. Immunol.* **2023**, *14*. 1090898.
